# European Society for Gynaecological Endoscopy (ESGE) Good Practice Recommendations on surgical techniques for removal of fibroids: part 1 abdominal (laparoscopic and open) myomectomy

**DOI:** 10.52054/FVVO.16.3.041

**Published:** 2024-09-30

**Authors:** E Saridogan, L Antoun, E.V.A. Bouwsma, T.J. Clark, A Di Spiezio Sardo, J Huirne, T.S. Walker, V Tanos

**Affiliations:** Elizabeth Garrett Anderson Institute for Women’s Health, University College London and University College London Hospital, United Kingdom; Birmingham Women’s & Children’s Hospital, University of Birmingham, United Kingdom; Department of Obstetrics & Gynecology, St. Antonius Hospital, The Netherlands; Obstetrics and Gynaecology Department, Università degli Studi di Napoli “Federico II”, Italy; Department of Obstetrics & Gynaecology, Amsterdam University Medical Center and Amsterdam Reproduction and Development Research Institute, The Netherlands; Gynaecology Department, Royal Cornwall Hospital, United Kingdom; Department of Basic and Clinical Science, University of Nicosia Medical School and Aretaeio Hospital, Cyprus

## Abstract

Uterine fibroids are the most common benign tumours of the female reproductive tract and can cause a range of symptoms including abnormal uterine bleeding, pain, pressure symptoms and subfertility. Surgery may be required for some symptomatic fibroids via abdominal or transvaginal routes. The European Society for Gynaecological Endoscopy Uterine Fibroids Working Group developed recommendations based on the best available evidence and expert opinion for the surgical treatment of uterine fibroids. In this first part of the recommendations, abdominal approaches to surgical treatment of fibroids including laparoscopic, robot- assisted and open myomectomy are described.

## Introduction

Uterine fibroids (also known as myomas or leiomyomas) are the most common benign tumours of the female reproductive tract and can be found by ultrasound in about 70-80% of women by the time of the menopause (Baird et al., 2003). Although some fibroids may be completely harmless, others may cause significant symptoms requiring surgical treatment. The European Society for Gynaecological Endoscopy (ESGE) Uterine Fibroids Working Group prepared two documents about surgical techniques to remove uterine fibroids without removing the uterus (i.e. myomectomy). This first document describes the surgical techniques for the removal of uterine fibroids via the abdominal route utilising either laparoscopic or open surgical methods. The second document covers hysteroscopic procedures (sometimes referred to as transcervical procedures) for fibroid removal. In this first paper, the term abdominal myomectomy is used to cover both laparoscopic and open procedures. Robotic myomectomy is included within the laparoscopic myomectomy section.

### Fibroid classification

There are two widely used classification systems for fibroids, one developed by the European Society for Gynaecological Endoscopy (ESGE) and the other adopted by the International Federation of Gynaecology and Obstetrics (FIGO) (Munro et al., 2011; Wamsteker et al., 1993). The FIGO classification is relevant to abdominal myomectomy because it adopted and expanded the initial ESGE classification that was restricted to submucosal fibroids. Both FIGO and ESGE classifications of uterine fibroids are shown in (Table I).

**Table I:** FIGO and ESGE classifications of uterine fibroids (modified from Munro et al., 2011; Wamsteker et al., 1993).

Fibroid type	ESGE Classification	Description	FIGO Classification	Description
Submucosal	0	No myometrial involvement – pedunculated	0	Pedunculated in the cavity
	I	<50% myometrial involvement	1	<50% intramural
	II	≥ 50%myometrial involvement	2	≥50% intramural
Intramural	-		3	Contacts endometrium, 100% intramural
			4	100% Intramural
Subserosal	-		5	Subserous and ≥50% intramural
			6	Subserous and <50% intramural
Pedunculated	-		7	Subserous pedunculated
Other	-		8	Cervical/parasitic
Hybrid leiomyoma	-		2-5	Submucous and subserous, each with less than half the diameter in the endometrial and peritoneal cavities

The “STEPW” or “Lasmar” hysteroscopic myoma classification was developed in 2005 (Lasmar et al., 2012). This classification not only evaluates the proportion of myometrial involvement as described in the original ESGE submucosal fibroid classification (Wamsteker et al., 1993), but also describes three additional parameters: the size, location in the cavity (topography) and extension of the base. This more detailed classification of submucosal fibroids may further help assess the feasibility of hysteroscopic myomectomy, informing clinical decision-making, patient counselling and surgical preparation (See Part 2 ESGE Good Practice Recommendations on Surgical Techniques for Removal of Fibroids: Part 2 Hysteroscopic Myomectomy, in press).

### Clinical assessment

Many fibroids are asymptomatic, but up to 50% cause symptoms that warrant therapy (Lethaby et al., 2017). Symptoms include anaemia caused by heavy menstrual bleeding, pelvic discomfort or pain, ‘bulk’ pressure symptoms and reduced fertility (Lethaby et al., 2017). The clinical assessment of women with fibroids and treatment options are summarised in (Table II).

**Table II:** Fibroids assessment and treatment options.

History	
General gynaecological history Focus on fibroid-related symptoms (see below) and their impact on reduced quality of life	
Symptoms	
Abnormal uterine bleeding Heavy menstrual bleeding Intermenstrual bleeding (IMB) Bulk, pressure symptoms Abdominal and pelvic pain (e.g. menstrual and non-menstrual pain/discomfort, dyspareunia) Abdominal distension Urinary and bowel symptoms (e.g. frequency, retention) Reproductive problems Infertility Miscarriage / preterm birth	
Examination	
Abdominal and bimanual pelvic examination to assess the size, composition and mobility of the fibroid uterus Consider vaginal speculum examination based upon bleeding / discharge symptoms and smear history	
Investigations	
Blood tests Full Blood Count (Hb+MCV), ferritin, iron binding capacity Imaging Transvaginal (TVS) / optional addition of transabdominal (TAS) ultrasound in case of large uterus MRI (for example if TVS/TAS inadequate (e.g. large and/or multifibroid uterus/ coexistence of adenomyosis) or to provide more information about fibroid vascularity, necrosis and proximity to adjacent organs. Hysteroscopy +/- biopsy Indicated if endometrial hyperplasia or cancer suspected Consider to aid hysteroscopic surgical planning (technique, equipment, need for down-regulation, multi-stage procedures etc.) for submucosal fibroids	
Treatment options*	
Medical	Non-steroidal anti-inflammatory drugs (NSAIDS) Tranexamic Acid (TXA) Combined oral contraceptive pill (COCP) Progestogens (local (Levonorgestrel releasing intrauterine system (LNG-IUS) and systemic (oral / parenteral) Gonadotrophin releasing hormone analogues (GnRHa) Pre-operative use to reduce fibroid volume Prolonged use (>3-6months) to manage symptoms in conjunction with add-back hormone replacement therapy (HRT) Oral GnRH antagonists (including combination therapies containing HRT) Selective progesterone receptor modulators (SPRMs) Liver function should be monitored before, during, and after treatment courses
Surgical	Myomectomy: Hysteroscopic or abdominal (Open or laparoscopic including robotic) Hysterectomy
Non-surgical	Uterine artery embolisation (UAE) Ablation (Transabdominal (laparoscopic guided) or transvaginal/transcervical (ultrasound guided) radiofrequency ablation or MRI guided focused ultrasound ablation)

Hb = Haemoglobin, MCV = Mean Corpuscular Volume, MRI = Magnetic Resonance Imaging

* No treatment may be necessary for minimal or no symptoms.

## Materials and methods

Electronic bibliographic databases (Medline / Embase) and the Cochrane Central Register of Controlled Trials from inception up to January 2024, were searched for randomised controlled trials, observational studies and expert opinions which addressed the surgical treatment of fibroids either in isolation or in comparison to medical and other non-surgical treatments. In addition, the National Institute for Health Research and Clinical Excellence (NICE) guidelines, the American Association of Gynecologic Laparoscopists (AAGL) practice reports, the European Society for Gynaecological Endoscopy (ESGE) guidelines and the British Society of Gynaecological Endoscopy (BSGE) statements that included any reference to the management of fibroids were reviewed.

The suitability and safety of surgery will vary according to patient factors, including patient preferences and the outcomes they desire as well as the characteristics of the fibroid(s) themselves. Management should thus be individualised. Moreover, the feasibility and efficacy of the surgical techniques described here may vary according to several factors that include background factors such as the woman’s age and comorbidities, her symptoms (e.g. pain, pressure symptoms, infertility), the primary aim of the treatment (e.g. eliminating/ improving bulk symptoms, treating abnormal uterine bleeding, reducing pain or enhancing fertility), the fibroid characteristics (localisation, appearance, number, size(s)), tubal status and history of previous surgery (i.e. recurrence). These factors will need to be taken into consideration when making a decision for surgery and selecting the most appropriate surgical technique.

### Defining abdominal myomectomy

Abdominal myomectomy includes both laparoscopic (including robot assisted) and open myomectomy. The first open myomectomy was reported in the 19th century and it remained the main option for fibroid removal until 1970s when the first cases of hysteroscopic and laparoscopic myomectomy were described (Semm, 1979). Hysteroscopic approaches, indicated for submucosal fibroids and fibroids abutting the endometrium (FIGO type 0-3 fibroids (Munro et al., 2011)) will be covered in “Part 2: Hysteroscopic Myomectomy”. An abdominal approach is indicated for some large (>3-5cm) FIGO type 2-3 fibroids and all FIGO types 4-7 fibroids require an abdominal approach for removal. Conventionally this has involved performing a laparotomy, but increasingly laparoscopic methods are being utilised (Dubuisson et al., 2016). More recently, robotic surgery has increased the feasibility of laparoscopic approaches to fibroid removal. This is potentially because myomectomy is suture-intensive surgery and one of the key advantages of the robotic-assisted surgery is the ease of suturing (Lee et al., 2021).

### Pre-operative planning and preparation

Fibroid mapping is essential to plan the peri- operative surgical approach and to determine the required pre-operative work-up (Munro et al., 2011). Pre-operative work-up includes the assessment of the number, size and localisation of the fibroids, their relation to the uterine cavity and tubes and their vascularisation (Munro et al., 2011). This is useful in predicting difficulties during surgery such as fibroids with protrusion towards the broad ligament and close relation to the uterine arteries or ureters, and development of postoperative intra-uterine adhesions. It also helps estimating the fertility prognosis when a myomectomy is performed for reproductive reasons. When the fertility chances are very low because of other reasons, for example poor ovarian reserve, damaged fallopian tubes or male factor issues, it is essential to discuss the likely benefit, if any, of abdominal myomectomy from a fertility perspective with patients before surgery. This includes discussing the potential negative effect of delay in commencing natural or assisted conception imposed by the need for recovery and healing to restore uterine integrity over several months after a myomectomy. This is especially important in older women and in women with low ovarian reserve. In these situations, patients may want to consider cryopreservation of their oocytes or embryos (Somigliana et al., 2008). Similarly, patients should be counselled about the relative pros and cons of a myomectomy compared with alternative, more conservative non-surgical treatments, to alleviate fibroid-associated symptoms such as abnormal bleeding and bulk, pressure effects. The cons for a surgical myomectomy includes the potential need for a planned caesarean section due to concern of scar dehiscence or uterine rupture and higher risk of abnormal adherence of the placenta in case of large and deep myometrial incisions.

#### Treating pre-operative anaemia

Significant fibroids often cause heavy menstrual bleeding leading to iron deficiency anaemia. Moreover, myomectomy carries a risk of significant blood loss during and after surgery. In a retrospective study comparing surgical outcomes by route of myomectomy in 575 women, it was found that 6.5% of patients undergoing an open abdominal approach and 1.1% of patients undergoing a minimally invasive approach (laparoscopic and robot-assisted) required blood transfusion in the perioperative period (Barakat et al., 2011). A Cochrane review, comparing surgical results after myomectomy by open surgery versus myomectomy by laparoscopy and hysteroscopy, could not draw any consistent conclusions on differences in blood loss according to these different approaches (Bhave Chittawar et al., 2014).

In addition to the risk of operative blood loss, it is important to treat pre-operative anaemia because pre-operative anaemia increases postoperative morbidity. A large retrospective cohort study among 12,836 patients who underwent gynaecological surgery (Richards et al., 2015) demonstrated that preoperative anaemia was independently associated with an increased odds of 30-day mortality (OR: 2.40, 95%CI: 1.06–5.44) and composite morbidity (OR: 1.80, 95%CI: 1.45–2.24). This was reflected by significantly higher adjusted odds of almost all specific morbidities including respiratory, central nervous system, renal, wound, sepsis, and venous thrombosis. This risk associated with preoperative anaemia did not appear to be corrected by use of perioperative transfusion (Richards et al., 2015).

As preoperative anaemia is easily detectable, it can be considered as a potentially preventable risk factor in patients undergoing elective surgery (Spahn et al., 2012). Therefore, haemoglobin levels of patients should be evaluated some time in advance before the planned surgery, in order to allow time for the pre-operative treatment of anaemia. This may involve oral or parenteral iron supplementation, blood transfusion and suppression of uterine bleeding using hormonal treatment with progestogens, GnRH analogues or SPRMs.

#### Pre-operative reduction in fibroid volume

Agents that are known to reduce fibroid volume are Gonadotrophin releasing hormone agonists (GnRHa), the selective progesterone receptor modulator (SPRM) Ulipristal Acetate (de Milliano et al., 2017), or GnRH antagonists (Huirne et al., 2001). The most studied agents for this indication are GnRH-agonists. There is clear evidence from randomised controlled trials (RCTs) that preoperative GnRHa can reduce both uterine and fibroid volume and improve haemoglobin levels and reduce peri-operative blood loss, although at the expense of increased oestrogen deficient side effects such as hot flushes, before surgery. Rates of more morbid and less cosmetically desirable vertical incisions and blood loss are also reduced in women undergoing an open myomectomy (Lethaby et al., 2017). Reduction in uterine volume may also increase the feasibility of minimally invasive, laparoscopic approaches and reduce the presence of intra-abdominal adhesions. In addition, there is no compelling evidence to support the notion that the use of GnRHa impact adversely on the identification of cleavage planes (de Milliano et al., 2017).

Administration of systematic GnRH antagonists prior to laparoscopic myomectomy appears to reduce peri-operative blood loss (Huirne et al., 2001) although data are mainly from small cohort studies and so there remains insufficient evidence to support the routine use of GnRH antagonist prior to myomectomy (Huirne et al., 2001). Oral GnRH antagonists have recently become available (Al-Hendy et al., 2021; Donnez et al., 2022) and most are combined with HRT to reduce the side effects. So far no clinically meaningful volume reductions have been found with GnRH antagonist combination preparations (de Lange et al., 2024).

Another pre-treatment agent is Ulipristal acetate. This oral drug is effective in the reduction of menstrual blood loss and in increasing pre-operative haemoglobin levels. However, in a double-blind randomised controlled trial comparing Ulipristal with GnRHa pre-treatment, GnRHa was in favour in terms of stronger volume reduction, reduction in fibroid vascularity, less peri-operative blood loss and surgical ease including better cleavage plane (de Milliano et al., 2017). Very recently, the risk- efficacy balance of Ulipristal has been investigated by the European Medicine Agency because it may cause drug-induced liver injury (DILI). After the review, they suspended Ulipristal from the marketing authorisation for the indication of pre- operative treatment for fibroid surgery (Middelkoop et al., 2021). National Institute for Health and Care Excellence (NICE) guidelines (National Institute for Health and Care Excellence, 2021) advised that it should only be used before menopause and when surgical procedures (including uterine fibroid embolisation) are not suitable and only after a thorough discussion with the patient about risks and benefits.

#### Patient selection

The location, number and size of the fibroids are essential in determining the procedure type and technique. Fibroid mapping using high-quality transvaginal ultrasound scan or MRI can help to assess the feasibility of performing the procedure laparoscopically or via laparotomy (Saridogan, 2016). The position of the fibroids in relation to the uterine arteries and fallopian tubes is also important, as well as the depth of penetration within the myometrium. Laparoscopic removal of fibroids deeply embedded in the myometrium may be associated with more blood loss than removal of more superficial fibroids and closing the uterine defect adequately requires better technical skills. Removing fibroids that impinge on the fallopian tubes or uterine vessels requires meticulous dissection and suturing (Guo and Segars, 2012; Parker and Rodi, 1994).

Myomectomy, either open or laparoscopic, is a surgical treatment particularly suited for those women who wish for future fertility or want to preserve their uterus for other reasons (Saridogan, 2016). It seems clear that, in well-trained and experienced hands, well-selected patients can have myomectomy performed via laparoscopy. Large, multiple or cervical fibroids may not be as suitable for a laparoscopic approach but are still amenable to a uterine conserving procedure via laparotomy that is facilitated by a number of preoperative and intraoperative measures aimed to minimise or replace operative blood loss (Parker and Rodi, 1994). In the past years experienced endoscopists are also able to take the pre- and intra-operative measures during laparoscopy or robotic surgery. Laparoscopic techniques should provide appropriately selected women with a safe and effective uterine-conserving procedure with possibly reduced post-operative morbidity and enhanced recovery.

Evidence from randomised control trials suggest similar cumulative pregnancy and live birth rates in women with otherwise unexplained infertility following laparoscopic versus open myomectomy (Palomba et al., 2007).

#### Informed consent

Obtaining appropriate consent is essential before surgery. The patient should be fully informed of all possible risks associated with the surgical procedure, including general risks of laparoscopic and open surgery, risks of intra-uterine/ abdominal adhesion formation, damage to the Fallopian tubes and potential risks during subsequent pregnancy and delivery (Orlando et al., 2021). Alternatives should be discussed. Surgery should only be performed when sufficient expertise is available in the centre by a surgeon and team with sufficient expertise and otherwise the woman should be referred to a centre with the required expertise (Johnson et al., 2013).

#### Pre-operative Antibiotics

Broad-spectrum antibiotics are indicated to reduce infective morbidity prior to laparotomy (World Health Organization, 2018). Antibiotics are widely used for major gynaecological procedures, but there is no significant evidence to say that their use specifically reduces myomectomy related infections (Spahn et al., 2013). There has been some evidence demonstrating lower surgical site infections in open myomectomies, but this was not translated across for the laparoscopic approach (Lethaby et al., 2017; Kim et al., 2019; Van Eyk et al., 2012; Banerjee et al., 2020).

### Interventions to reduce blood loss

Injection of synthetic vasopressin into the myometrium and/or broad ligament is effective in reducing blood loss during myomectomy (Kongnyuy and Wiysonge, 2014; Takeuchi et al., 2003) but can result in intraoperative hypertension and vasoconstriction. Whilst it is widely used during laparoscopic and open myomectomy in many countries, it is not available in some countries due to safety concerns. There is moderate evidence to support the use of vaginal misoprostol to reduce blood loss during laparoscopic and/or open myomectomy (Kongnyuy and Wiysonge, 2014) and the use of peri-cervical tourniquet by Foley catheter at open myomectomy in combination with temporary occlusion on the ovarian vessels (Alptekin and Efe, 2014). Only low-quality evidence supports interventions such as bupivacaine plus epinephrine, tranexamic acid, gelatin-thrombin matrix, ascorbic acid, dinoprostone, loop ligation and a fibrin sealant patch to reduce intra-operative blood loss (Hickman et al., 2016; Kumakiri et al., 2012; Opoku-Anane et al., 2020). There is no clear evidence to support the routine use of oxytocin or temporary vascular occlusion of the uterine / utero- ovarian vessels using clips or clamps (Kongnyuy and Wiysonge, 2014; Younes et al., 2022).

Cell Salvage / Saver should be considered in patients who are anaemic (Hb < 100 g/L) at the start of a procedure, who are expected to have prolonged surgery due to removal of multiple fibroids or who decline blood products (but will accept cell salvage). However, it is difficult to predict when reinfusion of cell salvaged blood is required and therefore, on a financial and sustainability basis, routine use is not recommended (Kumakiri et al., 2012; Son et al., 2014).

### Interventions to reduce adhesion formation

The development of adhesions can be considered as a physiological reaction following surgical exposure (Torres-de la Roche et al., 2023). In a systematic review with statistical pooling the formation rate, distribution and severity of postoperative adhesions in patients undergoing abdominal surgery was estimated using 25 studies in which the formation of adhesions was confirmed by “second-look” laparoscopy or laparotomy (Okabayashi et al., 2014). In patients who underwent gynaecological laparoscopic myomectomy, the weighted mean adhesion formation rates were 57% (95 % CI 20– 94) (Okabayashi et al., 2014).

The consequences of adhesion formation include chronic abdominal pain, dyspareunia and impaired fertility, small bowel obstruction and complications in further surgery, which can cause significant morbidity (Herrmann et al., 2020; Takeuchi et al., 2008). The Adhesions Research Special Interest Group of the European Society for Gynaecological Endoscopy (Torres-de la Roche et al., 2023; De Wilde et al., 2012) provided a list of anti-adhesion strategies, including shortening of operating time, meticulous haemostasis and improving intraabdominal environment by humidifying the CO2 and using lactated ringer’s solution for irrigation (De Wilde et al., 2012). In recent years, a number of barrier agents with different characteristics became commercially available such as oxidized regenerated cellulose, polytetrafluoroethylene surgical membrane, hyaluronic acid products, dual-sided hydrophilic film and fibrin sheet. Other strategies include the use of fluid and gel agents as well as pharmacological methods, aimed at modifying aspects of the healing process (Ahmad et al., 2020).

There are two recently updated Cochrane reviews on the effect of barrier agents and fluid and pharmacological agents for adhesion prevention in gynaecological surgery (de Milliano et al., 2017; Ahmad et al., 2020). Remarkably, in the review on barrier agents, none of the 19 included studies reported the outcomes of pelvic pain or live birth rate. The review on fluid and pharmacological agents included 32 included studies, of which one study reported on pelvic pain and three on live birth rate. Due to the lack of evidence and low quality of the studies sound conclusions could not be drawn. However, there is some limited evidence that suggests that using anti-adhesion measures such as oxidized regenerated cellulose, polytetrafluoroethylene surgical membrane, and hyaluronic acid products, may be beneficial in reducing postoperative adhesion formation. In a recent systematic review including eight RCTs focusing on the effectiveness of different adhesion barriers in the prevention of de novo adhesion development after laparoscopic myomectomy, adhesion barrier methods showing the most promising results were oxidized regenerated cellulose, hyaluronic acid products and, polyethylene glycol amine plus dextran aldehyde polymers (Borghese et al., 2021). In addition, hydroflotation and gel agents appear to reduce adhesion formation after gynaecological surgery, compared with no treatment (Koninckx et al., 2023; Torres-de la Roche et al., 2023). It should be mentioned that there is a large gap in evidence regarding actual effects on clinical outcomes, which are more important to women than the extent of their adhesions.

### Open myomectomy

Open myomectomy refers to the surgical removal of fibroids via a laparotomy. The procedure was first performed in 1840 and was the only uterus- preserving surgical option for the treatment of symptomatic fibroids until the late 20th century when laparoscopic and hysteroscopic techniques were developed (Saridogan., 2016). It is still a commonly performed procedure when there are multiple symptomatic fibroids that are less suitable for laparoscopic approaches. An open approach may also be considered, depending upon experience of the surgeon, where fibroids are located within more challenging locations such as the cervix or broad ligament.

#### Abdominal and uterine incision(s) and enucleation of fibroids

The abdomen is usually opened via a low transverse incision such as Pfannenstiel incision even for large uteri, because a mobile uterus can usually be mobilised and exteriorised through the abdominal incision. However, when the uterus is unusually enlarged (i.e. reaching the epigastrium), or the presence of significant adhesions posteriorly is anticipated, a midline incision may be required. Severe obesity may also restrict access to an enlarged uterus via a low transverse incision (Acién and Quereda, 1996).

Once the abdomen is opened, the pelvic and abdominal structures should be assessed. This may initially be difficult in the presence of fibroids as access to the pelvic cavity and visualisation may be compromised. In such cases the pelvic assessment can be carried out after removal of the fibroids. The uterus should be carefully examined, and the nature and location of the fibroids determined as well as their position in relation to the fallopian tubes and ovaries. Adhesions, if present, should be divided to restore normal anatomy. Exteriorising a large, mobile uterus through the laparotomy incision can help access to the fibroids, however this is not always immediately possible in case of large fibroids located at the level of the cervix or in the broad ligament. The choice of uterine incisions depends on the location of fibroids. In general, uterine incision sites are chosen to minimise the final number of incisions by giving access to maximum number of fibroids through each incision and avoiding the fallopian tubes, uterine and ovarian vessels and the bladder (Gehlbach et al., 1993). Although number of incisions may be less relevant that the total size and depth of the incisions.

It is important to expose the fibroid pseudocapsule, the plane between the fibroid and the myometrium, to facilitate smooth, atraumatic enucleation of the fibroids. Enucleation is achieved by a combination of traction and countertraction on the fibroid and myometrium as well as blunt and sharp dissection of the pseudocapsule. A number of instruments such as myomectomy screws and other clamps/forceps are frequently used to aid enucleation. Fibroids do not have a single vascular pedicle and the surrounding pseudocapsule houses the ‘vascular layer’. Hence remaining within the pseudocapsule tends to reduce the blood loss during enucleation. Where fibroids have undergone degeneration or infarction after UAE, the plane within the pseudocapsule can be less easy to delineate, resulting in piecemeal removal of tissue and increased risks of bleeding and myometrial trauma.

Smaller fibroids should be identified by meticulous palpation of the entire uterus/ myometrium and removed using an appropriate incision to reduce the risk of ‘residual fibroids’ (Gehlbach et al., 1993). Gentle manipulation and dissection during fibroid enucleation is important in fibroids abutting or protruding into the uterine cavity to minimise the risk of breaching the cavity.

Broad ligament and cervical fibroids are not only in close proximity to the uterine vessels but also to the ureters or may have altered their course. For this reason, the ureter should be carefully identified and dissected laterally, away from the fibroid for it to be removed from these locations (Saridogan, 2016). Identification of the ureter in this way minimises the risk of injury especially if bleeding ensues during fibroid removal because the uterine and other blood vessels within the broad ligament can be safely ligated.

#### Repair of the myometrial defect

Haemostasis is primarily achieved by effective closure of the myometrial defect, but targeted use of diathermy or vessel ligation may also be used in the presence of obvious bleeding vessels. Extensive coagulation of the myometrium should be prevented. Myometrial closure should ensure obliteration of all potential ‘dead space’ in the fibroid bed(s), but passing sutures through the endometrial cavity should be avoided to reduce the risk of intrauterine adhesions (Saridogan, 2016). Closing the defect in this way and avoiding excessive and indiscriminate application of diathermy allows for better healing of the myometrium and may minimise the risk of post-operative infection. Depending on the depth of incision, the myometrium may need to be repaired in multiple layers.

The serosa is then closed to achieve good approximation of the edges, covering exposed myometrium and giving additional haemostasis (Zhang et al., 2016). These measures may potentially reduce the adhesion formation around the uterus postoperatively. At the end of the procedure a thorough peritoneal lavage is carried out to remove any remaining blood to minimise risk of adhesion formation (Arung et al., 2011). Appropriate types of suture material for uterine closure are discussed in the laparoscopic myomectomy section below.

### Laparoscopic myomectomy

#### Positioning of patient and port placement

As in most gynaecological laparoscopic procedures, the patient is placed in a modified lithotomy position, with hands placed along the body, legs slightly flexed and abducted, and the pelvic floor protruding a few centimetres from the end of the operating table to allow easy mobilisation of the uterus, with an intrauterine manipulator (Tulandi and Einarsson, 2014). It is crucial to take measures such as anti-slip mats to prevent the patient sliding down towards the direction of the head when shifting in Trendelenburg position or during the course of surgery (Mattei et al., 2011; Sadashivaiah et al., 2018).

Three or four ports would usually be required for laparoscopic myomectomy, one of these would be later used for morcellation of the fibroid(s). Hence, it would be helpful to use a 11 or 12 mm port for this site and use it for driving needles in and out of the abdominal cavity during suturing. Port placement should allow enough space between the laparoscope and the uterus. Whilst an umbilical port would usually be selected for the laparoscope, using an epigastric port for the laparoscope would help in the presence of large fibroids or a large uterus. The other ports should be positioned according to the location of fibroid(s), type of incision(s) that would be made and the suturing technique of the surgeon. Surgeons who use ipsilateral suturing technique would place two lateral ports on the side they stand, whereas those who use contralateral suturing would use two ports on opposite sides. Some surgeons, however, prefer suturing through a lateral port and a suprapubic port (Saridogan., 2016).

To perform successful robot-assisted surgery, an experienced team is required to execute essential steps in preparing a patient for surgery; these are patient positioning, robotic port placement, and docking (Quaas et al., 2010). Four or five ports would be required depending on the number of robotics arms that would be used. The layout of port placement and size of the ports vary significantly depending on the robotic platform that is in use, as well as the surgeon’s preference. Similar to laparoscopic myomectomy, the camera port is usually placed either at or above the umbilicus depending on the size of the uterus and surgeon preference. This port accommodates the laparoscope which provides the three-dimensional imaging. Similar to laparoscopic myomectomy, with robotics as a general rule, at least a hands- breadth distance or approximately 8–10 cm between the laparoscope and the top of an elevated uterus or leiomyoma during manipulation is necessary in order to allow for an adequate working distance between the laparoscope and the uterine fundus. This is a critical concept during myomectomy because the enucleation process will result in the fibroid telescoping out toward the endoscope (Moawad et al., 2019). This loss of optical working distance must be compensated for at the beginning of the case or else instrument manoeuvrability will be compromised. Two additional ports that mount directly to the operating arms on the patient-side cart are placed to the left and right of the camera port, respectively. For larger uteri or leiomyomata, these ports are also moved more cephalad. A fourth trocar serves as an accessory port and can be placed between the camera port and either the left or right lateral ports (Moawad et al., 2019). This is used to facilitate introduction of sutures as well as instruments used for retraction and suction/irrigation (Senapati et al., 2015; Takmaz et al., 2018). An alternative placement for this accessory port is in either the left or right lower quadrant.

#### Uterine incision(s) and enucleation of fibroids

Uterine incisions to remove fibroids are tailored according to the location of fibroid(s) and the surgeon’s preference for suturing. As a general rule, incisions should be designed to avoid extending onto the Fallopian tubes, ovarian ligaments and main uterine arteries. Incisions can be made using ultrasonic instruments, monopolar needles, hooks or scissors, or laser. The incision should penetrate deep into the fibroid to identify the plane between the fibroid and the myometrium (Figures 1A and 1B). There is a tendency to remain too superficial leading to starting dissection into the overlying myometrium and this tends to make enucleation more difficult. The incision is then extended to the required length once the correct plane is identified. Fibroid enucleation is achieved by using a combination of traction and countertraction, as well as division of the fibroid from the pseudo capsule. For mechanical traction / counter traction, 5 or 10 mm instruments with strong grasping / fixation ability such as myoma screws, tenaculums, tooth graspers or claw forceps are used. For robot assisted myomectomy, a specially designed tenaculum can be used for this purpose.

**Figure 1:**
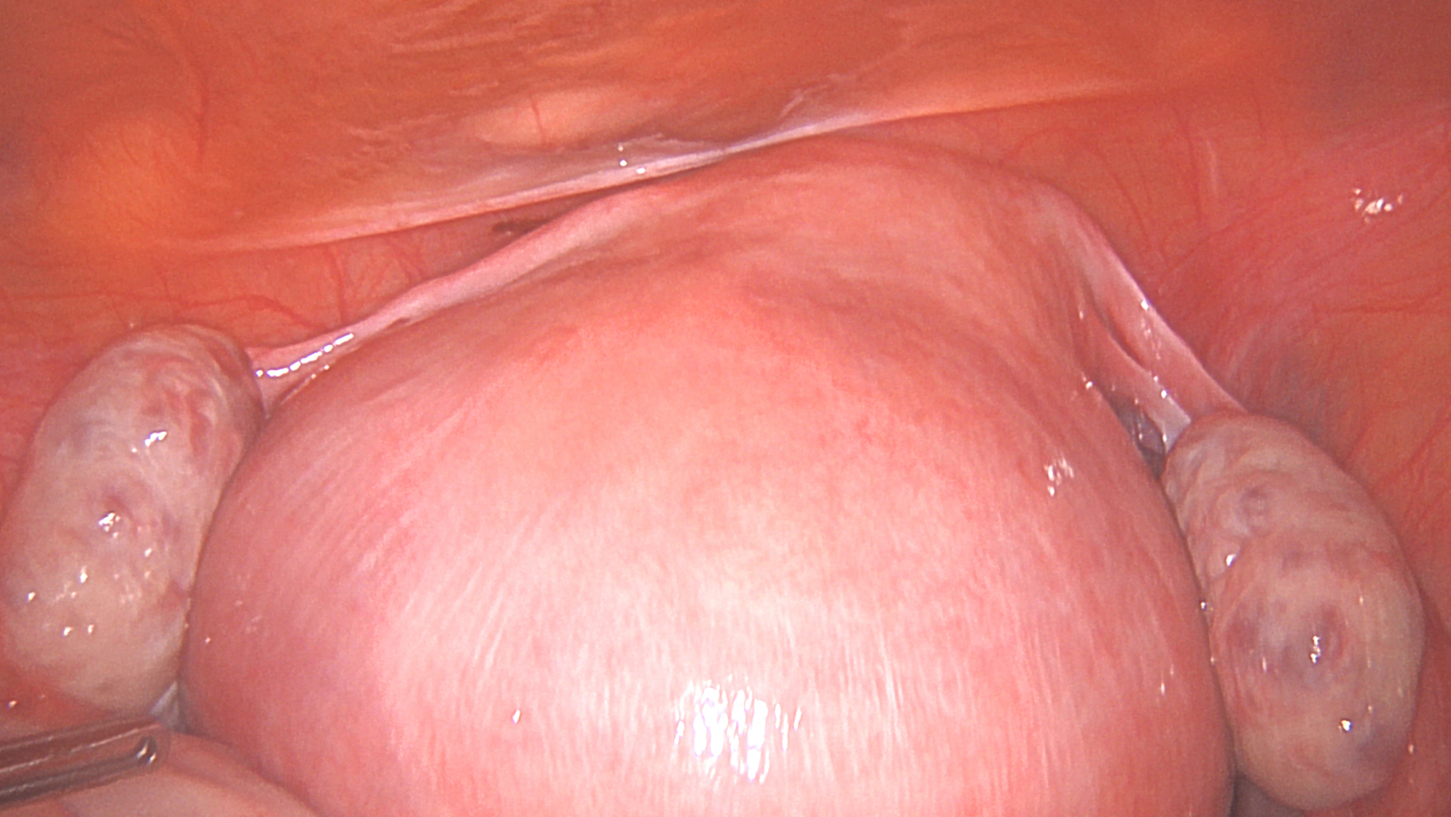
Surgical steps for laparoscopic removal of a large posterior wall fibroid. Figure 1A. A 9 cm fibroid protruding outwards on the posterior wall described as intramural by MRI.

**Figure 1B:**
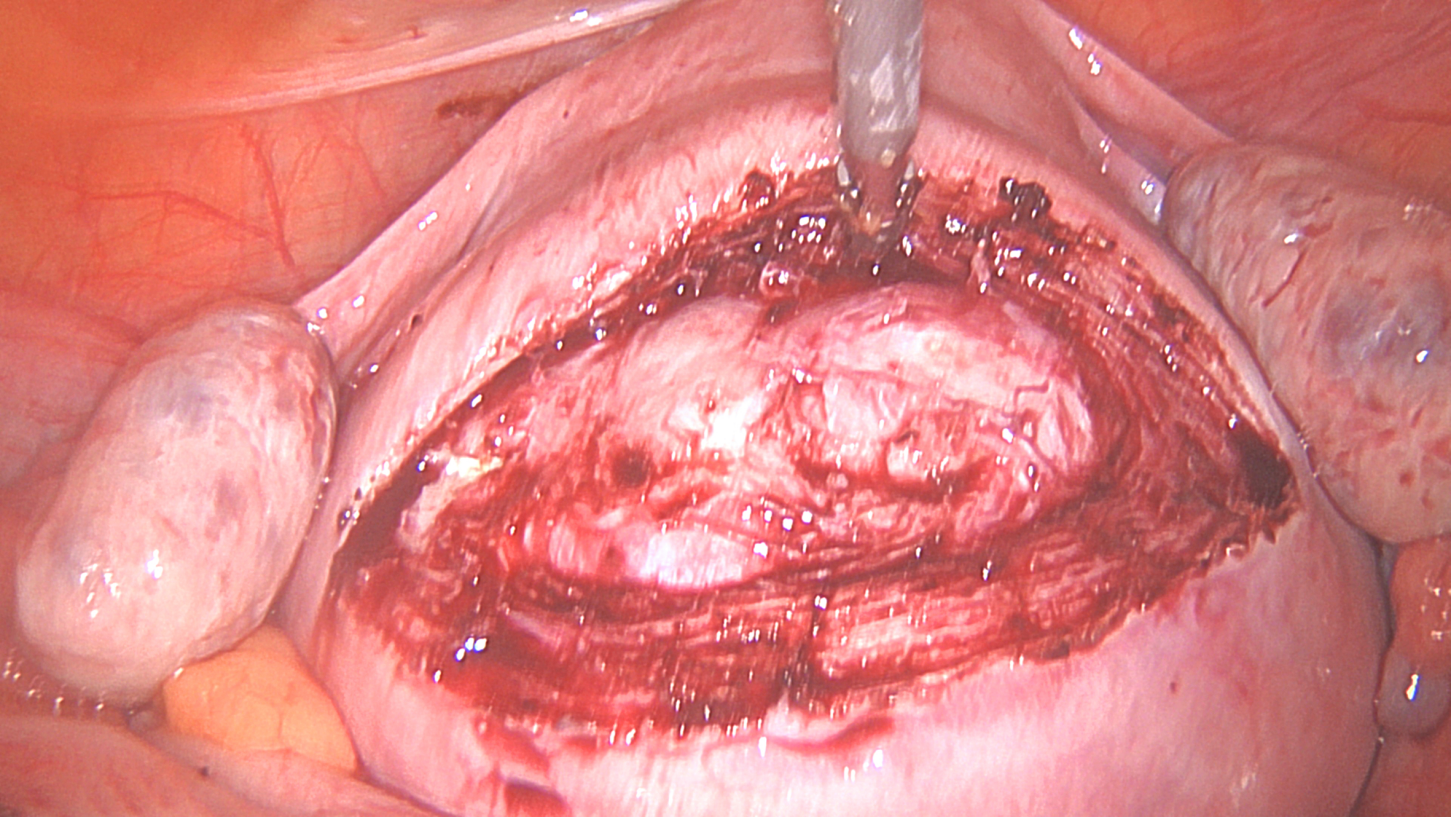
The incision over the fibroid to reach the fibroid before extending it to the desired length.

When approaching the base of the fibroid, dissection should be carefully performed to avoid pushing instruments into the uterine cavity, or avulsing the adherent endometrium, although opening the cavity may sometimes be inevitable when the fibroid has substantial protrusion into the cavity. Smaller fibroids should be placed in a preloaded endobag, in the Pouch of Douglas or sutured onto a string to avoid losing them amongst the loops of small bowel or beneath the omentum. Larger fibroids can be placed above the pelvic brim into the iliac fossae as they would be relatively easy to locate at a later stage (Moawad et al., 2019) (Figures 1C and 1D).

**Figure 1C:**
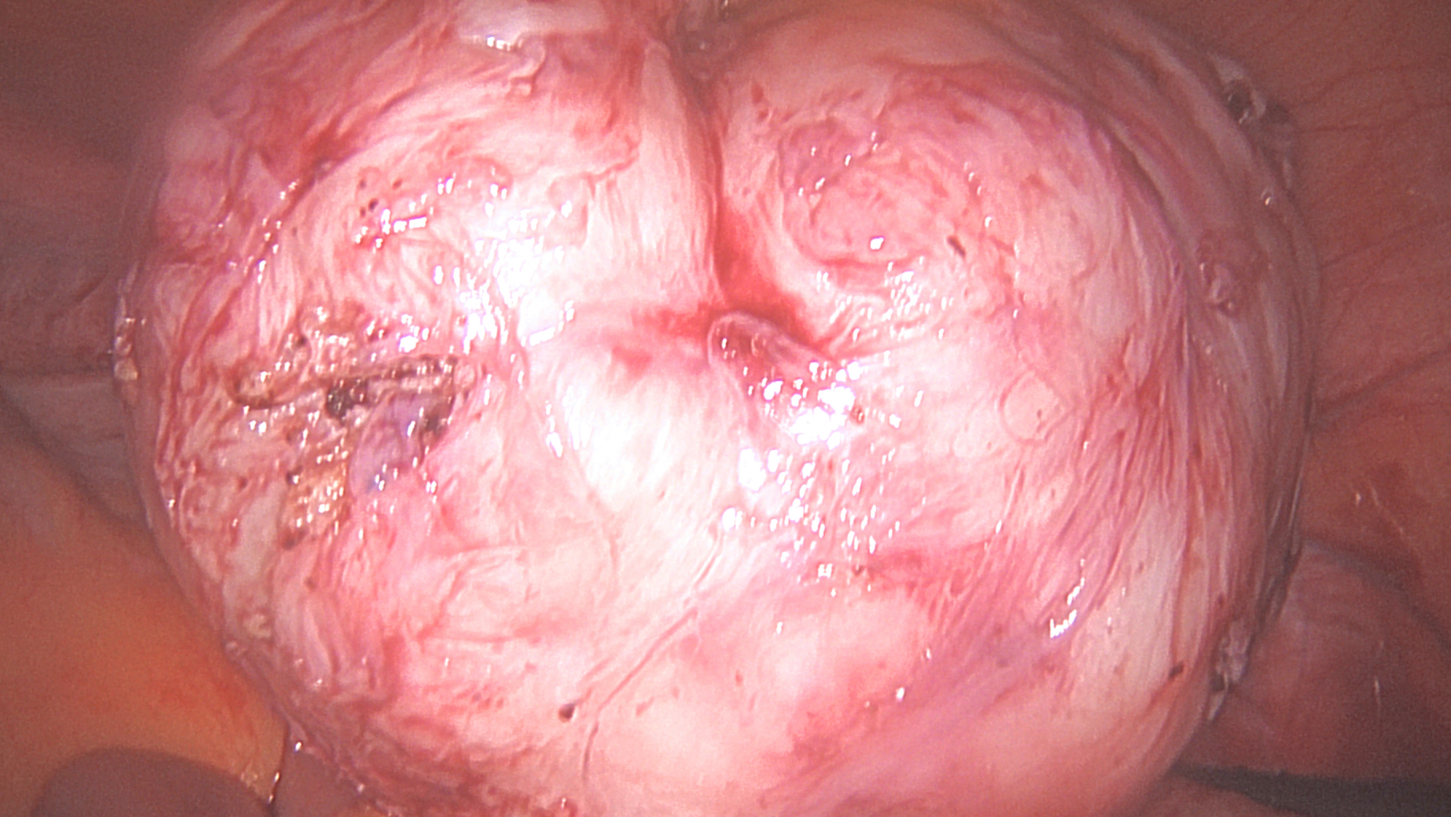
Removed fibroid.

**Figure 1D:**
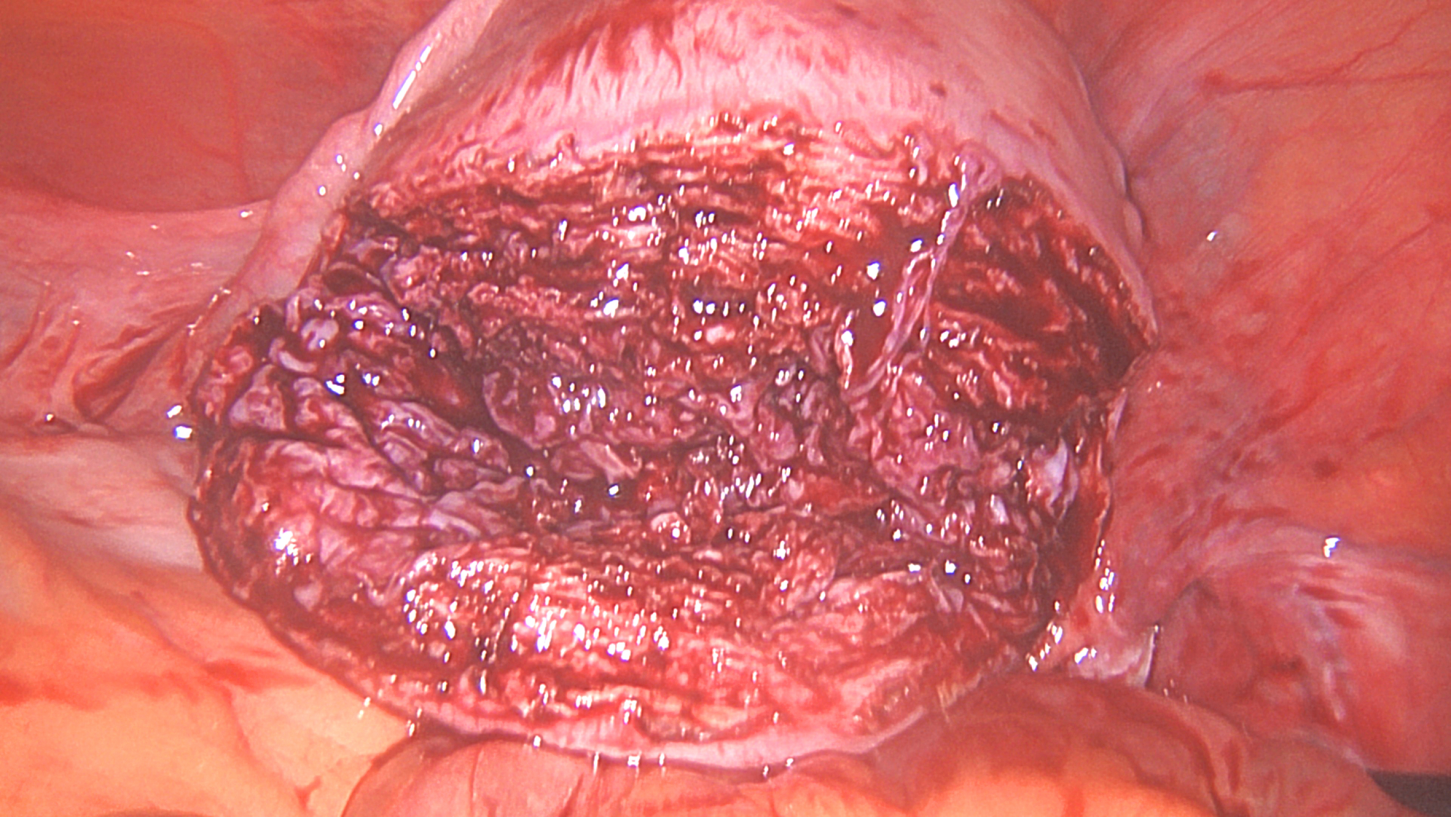
Uterine myometrial defect after fibroid removal.

Excessive haemostasis using diathermy should be avoided during and after enucleation of the fibroids, as this may increase the risk of uterine rupture in future pregnancies (Parker et al., 2010). Targeted diathermy for haemostasis can be performed for obviously bleeding vessels, but the dominant method of haemostasis remains suturing to repair the myometrium (see later).

#### Identification of the endometrial cavity

Abdominal myomectomy may cause intra-uterine adhesion formation that may compromise fertility outcomes and increase the potential risk of abnormal placentation and related complications (Bhandari et al., 2016). The risk of intrauterine adhesion development is substantially higher when the uterine cavity is opened during myomectomy (Şükür and Saridogan, 2021). Careful dissection of the fibroid to reduce likelihood of opening the endometrial cavity and avoiding unnecessary coagulation are highly relevant to preserve endometrial function. Intrauterine blue dye application in the uterus may facilitate early identification of the endometrium during a myomectomy (Bhandari et al., 2016). This is useful during both enucleation of the fibroid(s) and suturing the incision(s). An additional advantage of instilling blue dye into the uterus is that during the procedure the patency of the tubes can be tested if indicated. More recently the use of indocyanine green (ICG) has been described for this purpose (Ferreira et al., 2019).

#### Repair of the myometrial defect and choice of suture

The type and size of suture material and needles vary between the teams. The commonly used materials for both open and laparoscopic/robotic myomectomy include braided sutures such as polyglactin 910 or polyglycolide (Vicryl™, Velosorb™), monofilament sutures such as polypropylene, polydiaxanone, poliglecaprone or polyglyconate (Prolene™, PDS™, Monocryl™ or Maxon™) or barbed sutures (Alessandri et al., 2010; Stabile et al., 2021). The length of suture should be individualised to suit the technique, shorter suture lengths may allow easier suture handling and reduce entanglement with excessive suture (Fernandes et al., 2021).

The aim is to close the uterine defect created by the removal of the fibroid, in as many layers as is required without leaving ‘dead space’ as much as possible, whilst avoiding the uterine cavity (Fernandes et al., 2021) (Figures 1E, 1F and 1G). Leaving as little suture material exposed on the serosal surface may reduce the formation of adhesions, techniques such as a ‘baseball suture’ have been described to facilitate this (Fernandes et al., 2021). There is, however, no one technique and the key principle to close the layers and reduce the dead space.

**Figure 1E:**
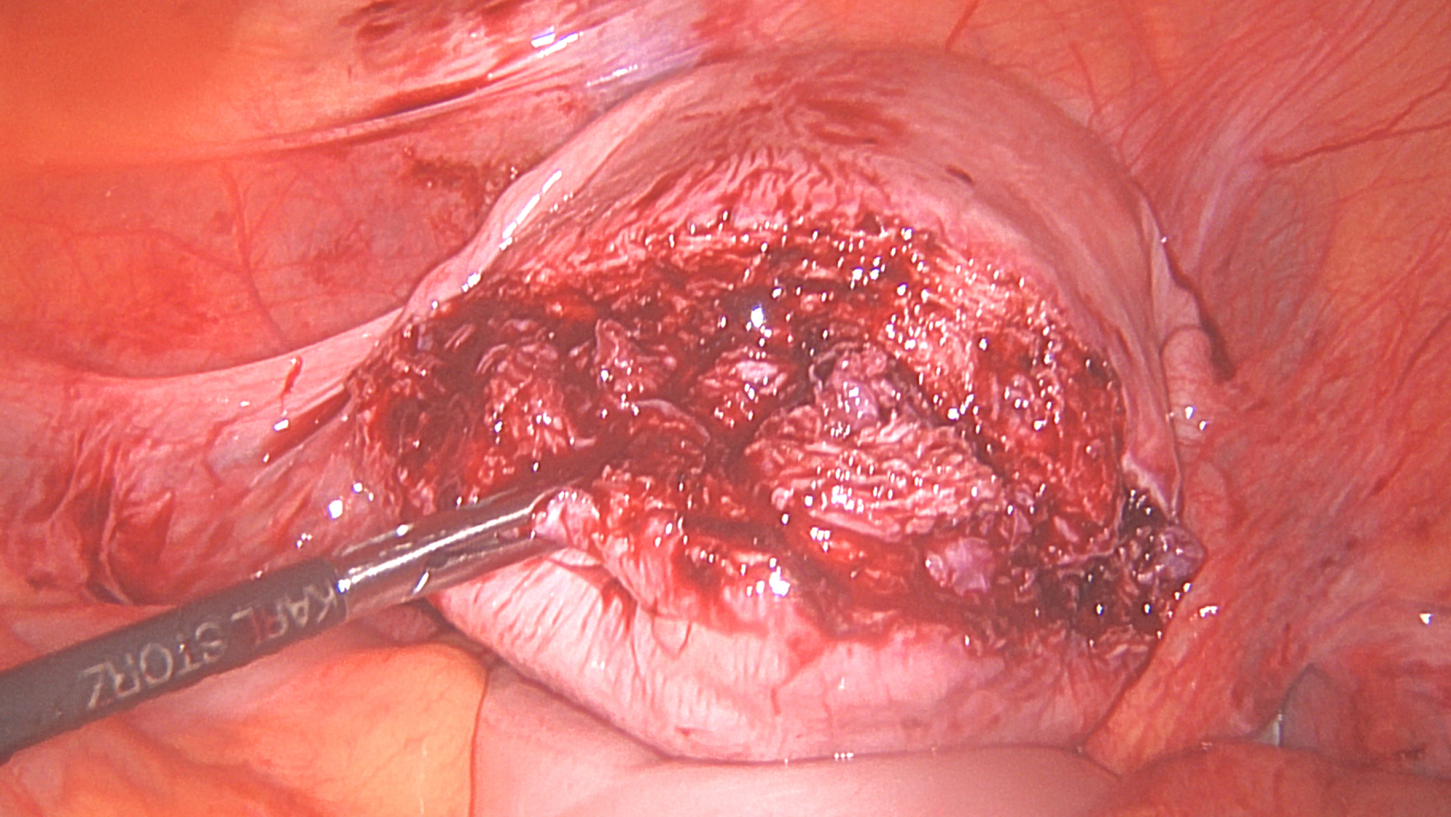
The first layer myometrial repair completed.

**Figure 1F:**
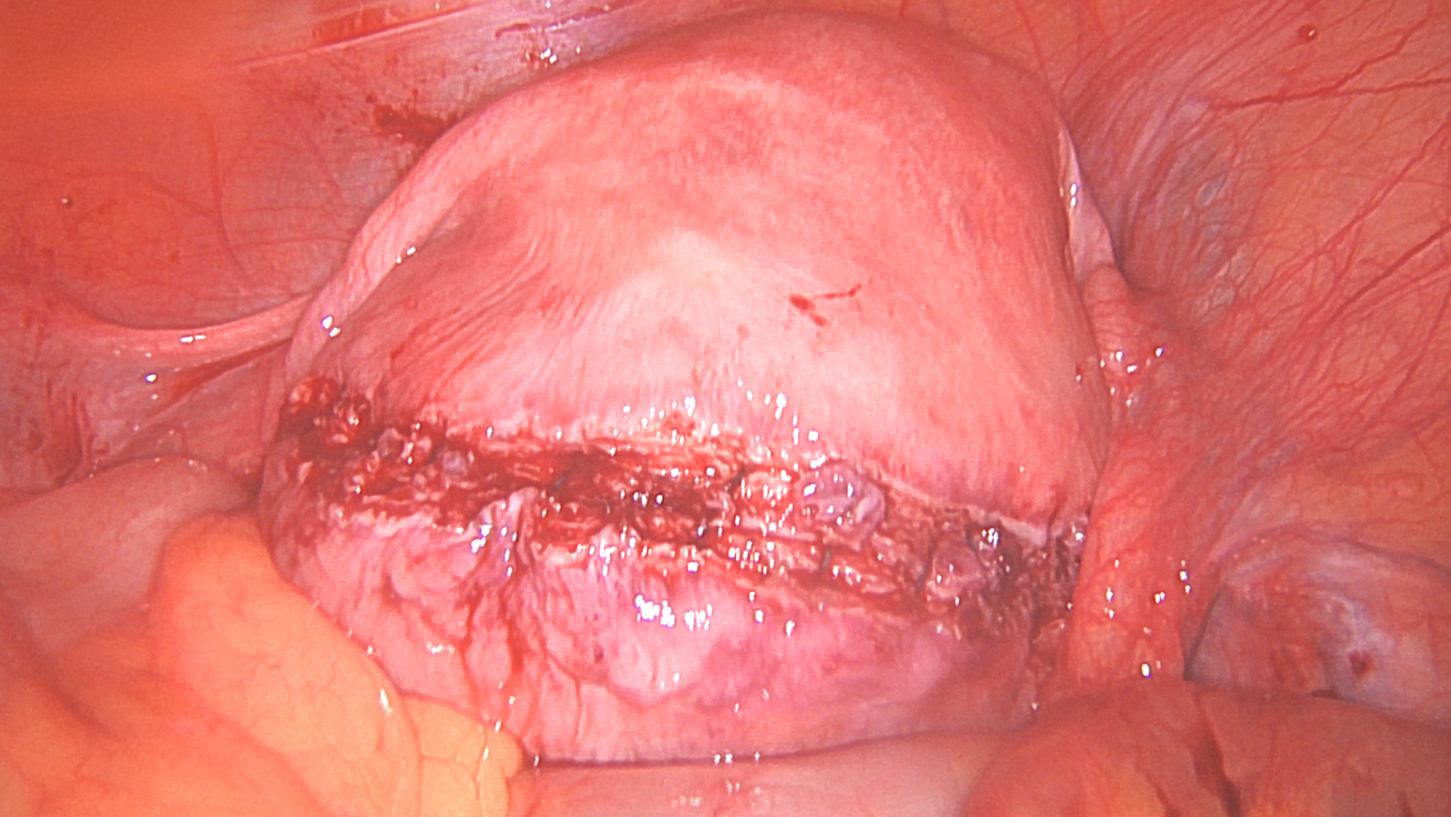
Second layer myometrial repair completed.

**Figure 1G:**
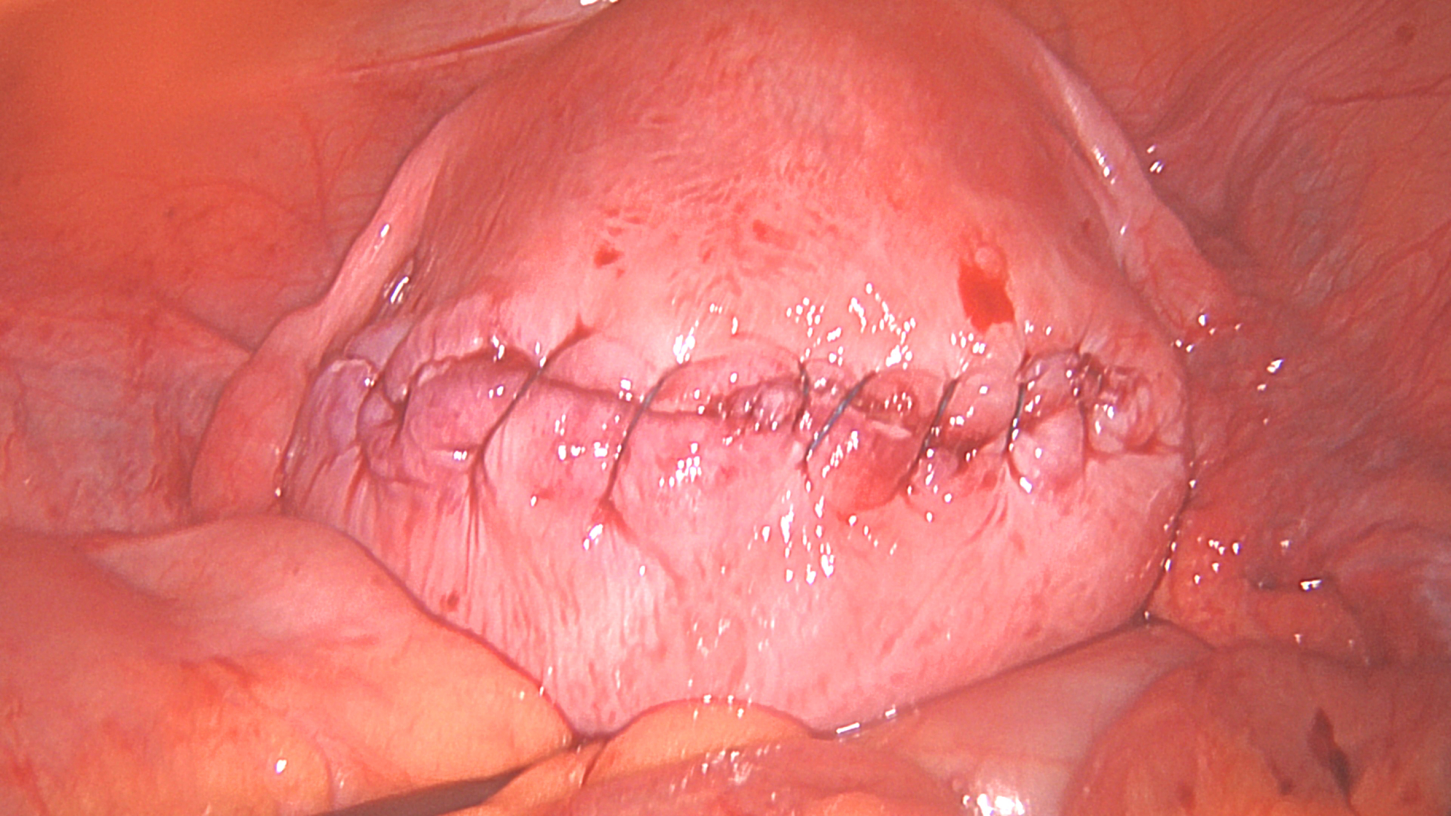
Serosal closure.

One concern is that conventional suturing can add additional time to the procedure and this in turn can mean potentially more blood loss. Barbed sutures appear to be quicker than conventional suturing, therefore reducing the operative time, this in turn may also reduce blood loss (Zhang et al., 2016). The use of knotless sutures may also allow an easier technique of tissue approximation and suturing and be advantageous for the surgeon (Tulandi and Einarsson, 2014).

However, it should be remembered when using barbed sutures that there have been case reports of bowel becoming adherent to the barbed suture extending from the wound and causing intestinal obstruction. These complications can potentially be reduced by reducing the amount of suture that is exposed, ensuring the ‘end’ of the suture is cut short and covering the suture line with one separate knotted suture and with an adhesion barrier (Alessandri et al., 2010; Stabile et al., 2021) (Figure 1H).

**Figure 1H:**
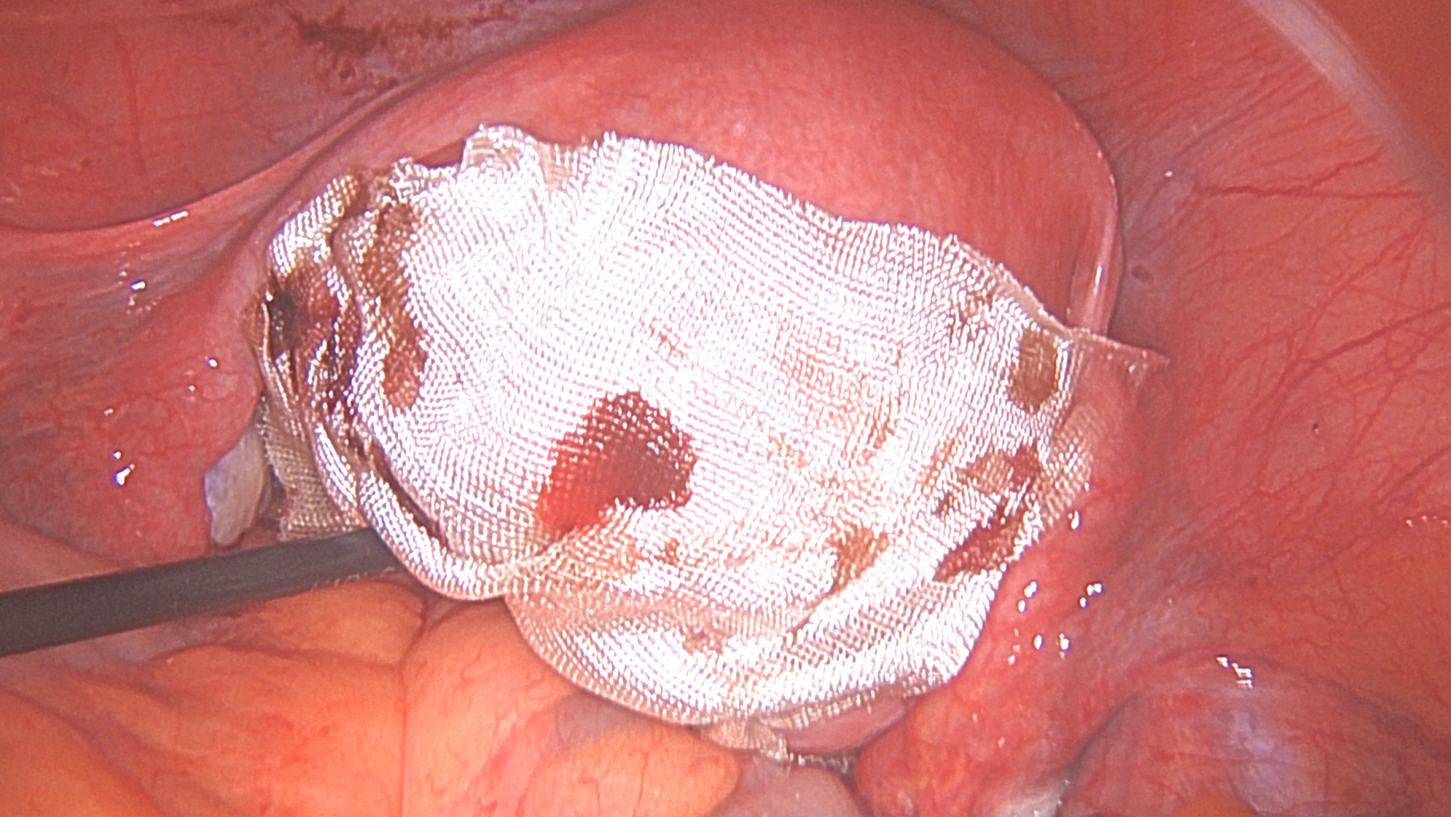
Second layer myometrial repair completed. G. Serosal closure. H. Incision and exposed barbed suture covered with a sheet of oxidised regenerated cellulose. (Pictures courtesy of Professor E. Saridogan)

#### Fibroid removal and morcellation

With the introduction of laparoscopic myomectomy, new techniques were developed to remove the specimen from the abdominal cavity. Morcellation is a procedure during which larger specimens are broken into smaller pieces, in order to remove them from the abdominal cavity (van der Meij et al., 2018). For this purpose, reusable and single use electromechanical morcellators were developed.

These usually involve a motor that powers a rotating blade which is housed in a hollow cylinder. The fibroid is pulled towards the blade with a heavy grasper or tenaculum and the morcellator is activated. The rotational action of the blade cuts a fibroid strip that is pulled into the hollow cylinder and then extracted out of the abdominal cavity. Presence of a ‘lip’ at the tip of the cylinder keeps the blade on the surface of the fibroid and allows ‘peeling’ style fibroid morcellation, enabling longer strips to be removed.

In 2014 possibility of spread of malignancy during the morcellation process was raised (Brölmann et al., 2015) and this led to significant changes in clinical practice including detailed preoperative assessment and counselling, in-bag morcellation (where the surgical specimen is inserted into the containment bag, the bag inflated with CO2 and tissue morcellation performed inside the bag) and abandoning electromechanical morcellation altogether in favour of manual morcellation by small incisions using the so called Chinese “paper roll” (Wong et al., 2010) or open surgery in the following years. These diverse approaches are still in practice to date.

In 2015, the ESGE carried out a systematic literature review in order to evaluate the risk of malignancy in presumed benign fibroids, potential risk factors and investigation methods (Brölmann et al., 2015). The overall risk of sarcomatous changes in the uterus from all published articles was 0.14 % (1 in 700). However, there are large differences between publications with figures varying from 0.49 % (1 in 204) (Takmaz et al., 2018) to 0.056 % (1 in 1,788) (Richards et al., 2015). On average, papers that look at myomectomy specimens give a lower risk of sarcomatous change of 0.08 % (1 in 1,306) compared to those that look at hysterectomy specimens where the overall pooled risk is 0.15% (1 in 650) (Senapati et al., 2015). The risk appears to be age related. Although the estimates vary widely, the following numbers for risk of sarcoma were derived for the Morcellation Consent Form of the Royal College of Obstetricians and Gynaecologists (2019) based on two main studies; 1:769-1250 for women < 50 years, 1:172-303 for women 50-59 years and 1:65-278 for women > 60 years based on study by Mao et al. (2015) and 1:304-574, 1:158-216, and 1:98-154 for the same age brackets respectively based on study by Brohl et al. (2015).

Limited data from relatively small randomised controlled trials does not show significant differences in safety and duration of morcellation between uncontained, in-bag electromechanical and in-bag manual morcellation approaches (Frasca et al., 2018; Venturella et al., 2016). However, it would be difficult to demonstrate a potential benefit on prevention of dissemination of sarcoma as these are very rare events. The data available suggest that in-bag morcellation does not prolong the surgical time significantly. It has the additional advantage that it prevents the development of peritoneal implants of fibroid tissue (Zullo et al., 2020).

### Complications of abdominal myomectomy

Intraoperative complications include bleeding, transfusion, damage to visceral organs or structures and hysterectomy. The latter is rare and is more likely to occur in the presence of multiple large fibroids or in cases of repeat myomectomy (Tanos et al., 2023; Kikuchi et al., 2008). High temperature and febrile illness on postoperative day 1 or 2 is quite common due to pyrogenic substances being released from the myometrium (Saridogan., 2016). This does not always require antibiotic treatment, but the possibility of infection should be considered and broad spectrum antibiotics started, especially if multiple uterine incisions were made, there was significant blood loss, the patient is unwell or the temperature remains high beyond the second postoperative day.

Other postoperative risks include pelvic abscess formation, development of a paralytic ileus, bowel obstruction and thrombosis. Adhesion formation in the long term is a common occurrence after myomectomy that may cause chronic pain, bowel dysfunction and subfertility, (Tanos et al., 2023; Kikuchi et al., 2008) 

The prevalence of complications depends on the complexity of cases included in the published literature. In a case series of 200 patients requiring open myomectomy with uterine size of 16 weeks or larger the overall major complication rate was 30% including major bleeding requiring blood transfusion (24.5%), visceral organ injury (2.0%), return to theatre (3.5%), readmission (2.0%) and hysterectomy (1.0%) (Pundir et al., 2013). The risk factors for major complications were uterine size of 20 weeks or larger, removal of 10 or more fibroids and operations requiring a midline incision. Both hysterectomies were in patients undergoing a repeat myomectomy and all other major complications were substantially more common in this group compared to primary open myomectomy.

The larger case series of laparoscopic myomectomy report major complication rates of 3-3.5% (Sizzi et al., 2007; Bean et al., 2017). The risk factors for complications were preoperative anaemia, larger fibroids, removal of three or more fibroids and lack of use of synthetic vasopressin (Sizzi et al., 2007).

Thromboprophylaxis for all major abdominal surgery needs to be considered. The measures include early mobilisation, graduated compression stockings, intermittent pneumatic compression and pharmacological agents such as low molecular weight heparins (LMWH). Pharmacological agents need to be weighed against risk of bleeding, if high risk for postoperative bleeding, mechanical prophylaxis should be continued until it is safe to switch to pharmacological agents. Scoring systems such as Caprini risk assessment tool or other similar formula to calculate risk and duration of treatment can be useful (Cronin et al., 2019). These tools take a number of factors into account including the patient’s age, body mass index, duration of surgery, smoking status and additional morbidity. A proportion of the cases with significant fibroids will need 7-10 days of prophylactic anticoagulation.

### Postoperative management

#### Postoperative recovery and convalescence advice

The rationale of minimally invasive techniques is that the use of small incisions is beneficial over conventional open surgery, as it results in less tissue trauma and inflammation, leading to reduced pain, faster mobilisation, shorter hospital stay and postoperative recovery time, translating into speedier return to normal activities and work (Bhave Chittawar et al., 2014; Aarts et al., 2015). With the adoption of enhanced recovery programmes, same day discharge appears to be feasible and safe in a significant proportion of patients after minimally invasive myomectomy (American College of Obstetricians and Gynecologists, 2018; Applebaum et al., 2023). The Cochrane review comparing minimal invasive techniques versus open myomectomy demonstrated that myomectomy by laparoscopy was a less painful procedure compared with open surgery with lower pain scores at 6 and 48 hours after surgery. In addition, patients in the laparoscopic group had a shorter median length of stay compared to women in the open surgery group (Bhave Chittawar et al., 2014). The review did not report on duration until return to normal activities or work but a prospective nationwide registry (COMPARE-UF) (Laughlin-Tommaso et al., 2020) has assessed the differences in recovery times between myomectomy routes. In a total of 1206 women who underwent a myomectomy (338 hysteroscopic, 519 laparoscopic, and 349 abdominal) in the United States (Laughlin-Tommaso et al., 2020). Return to usual activities averaged 0 days (IQR 0-14) for hysteroscopic myomectomy, 21 days (IQR 14-28) for laparoscopic myomectomy, and 28 days (IQR 14-35) for abdominal myomectomy.

Return to work averaged 4 days (IQR 3-10) for hysteroscopic myomectomy, 21 days (IQR 14-39) for laparoscopic myomectomy, and 42 days (IQR 28-56 days) for abdominal myomectomy.

Two smaller studies assessed recovery times in laparoscopic myomectomy. In a sample of 194 Canadian women the mean duration until return to office work was 12.5 days (11.4–13.7) and to physical work was 16 days (13.7–18.3) (Liu et al., 2010). In a study performed in the United Kingdom in a sample of 71 women who underwent laparoscopic myomectomy 39,4% of the women did not return to normal activities, including work, after 8 weeks (Huff et al., 2018).

These studies demonstrate that there is substantial variation in recovery times in women recovering from myomectomy, even within the same type of approach. Moreover, in some of these studies actual recovery times exceeded the recovery time considered appropriate by specialists. One explanation for the differences in recovery times between populations might be geographical differences in attitudes towards health and work as well as differences in the organisation of social and healthcare systems. Secondly, because of increasing experience in the technique, the complexity of cases being carried out laparoscopically has probably increased over time, with a resultant increase in the necessary convalescence time (Huff et al., 2018). In another field of benign gynaecologic surgery, it has been demonstrated that evidence-based convalescence advice plays a crucial role in determining the length of recovery of patients (Hollenbeck et al., 2008). Managing recovery expectations might be equally important in reducing recovery times as the degree of invasiveness of the surgery itself (Vonk Noordegraaf et al., 2014; Bouwsma et al., 2017; van der Meij et al., 2018). Therefore, to obtain full potential of minimal invasive techniques, it is recommended to invest in perioperative guidance and education of women undergoing myomectomy.

#### Second look hysteroscopy

The incidence of intrauterine adhesions following myomectomy varies widely from (1.07%-78%) (Bhandari et al., 2016; Gambadauro et al., 2012; Yang et al., 2008). Damage to the endometrium is considered to be a significant factor in the causation of intrauterine adhesions (Şükür and Saridogan, 2021). In cases where fertility is a primary concern and endometrial damage has occurred or been suspected during myomectomy, an ‘early’ hysteroscopy should be considered to exclude and treat any uterine adhesions that may have formed and could have an impact on the patient’s fertility (Sebbag et al., 2019). Performing as an outpatient procedure may be preferable to reduce the patient’s exposure to repeat general anaesthesia, however patient preference must be taken into account. There is no specific time to wait, but a minimum of 6 weeks would be sensible to allow time for tissue to heal and reduce the risk of injury to the suture line (van Wessel et al., 2021) whilst allowing for hysteroscopic division of filmy adhesions before denser fibrosis with greater cavity distortion sets in.

### Training and case load

To optimise clinical outcomes, the various types of myomectomies should only be performed in centres where there are surgeons and operating teams with sufficient expertise (Johnson et al., 2013). During the learning curve in laparoscopic myomectomy, a caseload of 100 patients was found to reduce complications rates and reduce hospital stay for patients (Shah et al., 2016). Surgeons performing specific types of myomectomies should maintain an adequate case-load to retain and enhance surgical skills, as higher volume surgeons generally achieve better outcomes (Shah et al., 2016).

### Alternatives to myomectomy

Non-surgical alternatives for the treatment of uterine fibroids (Table II) such as various ablation techniques (transcervical or laparoscopic radiofrequency ablation, MRI- or US- guided focussed ultrasound), uterine artery or fibroid embolisation or GnRH agonists or antagonists are beyond the scope of this document.

Currently there are insufficient data to advocate ablation techniques in women who want to preserve their fertility. It has been shown that transcervical uterine fibroid ablation does not induce intra- uterine adhesions (Bongers et al., 2019), but pregnancy rates post-treatment were not extensively studied. A recent review reported 30 pregnancies after laparoscopic radiofrequency ablation, this resulted in 26 full-term births, with a miscarriage rate of 13.3% (Berman et al., 2020). A recent systematic review concerning MRI or US guided focused ultrasound reported only 124 pregnancies. Pregnancy rates varied between 7 and 36% with a miscarriage rate of 39%. (Anneveldt et al., 2021). These results do not look very promising; however, caution is required as the studies excluded patients with a desire to conceive. But in general, we may conclude that with currently available evidence a myomectomy is the treatment of choice in women with a desire to conceive.

Uterine artery embolisation (UAE) is less suitable in women who wish to preserve their fertility since it is associated with lower pregnancy rates in comparison to a myomectomy and it may have a negative effect on the endometrium, ovarian reserve and in some cases, it may even induce early menopause (Gupta et al., 2014). It is also thought to increase miscarriage rates compared to expectant management (Homer and Saridogan, 2010).

## Conclusion

These ESGE Good Practice recommendations provide guidance to optimise the care of women undergoing surgical removal of uterine fibroids using an abdominal approach. Achieving optimal clinical outcomes necessitate an understanding of the indications for abdominal myomectomy, diagnostic work up and treatment planning as well as the importance of good surgical technique whether this be utilising laparoscopic, robotically assisted or open methods.

### Recommendations

1. Women with uterine fibroids should have an individualised management plan taking into account their preferences including fertility aspirations as well as the location, size and number of fibroids, Women should be counselled about all appropriate surgical and non-surgical options including their risks and benefits.
2. Pre-operative imaging with ultrasound or MRI to map and characterise the fibroids should be carried out to help plan abdominal myomectomy.
3. The surgical approach to abdominal myomectomy should be determined based upon the size, number and location of fibroids, the expertise of the operating surgeon / surgical team and patient preference.
4. Gonadotrophin-releasing hormone analogues should be considered preoperatively to reduce fibroid and uterine volume, help correct anaemia (in conjunction with iron supplementation) and reduce peri-operative blood loss.
5. For laparoscopic myomectomy the camera port placement should allow enough space between the laparoscope and the uterus, whether this be sited in the umbilicus or epigastrium. The other ports should be positioned according to the location of fibroid(s), type of incision(s) that would be made and the suturing technique of the surgeon.
6. The number of uterine incisions should be kept to a minimum and avoid extending onto the Fallopian tubes, ovarian ligaments and main uterine arteries.
7. Identification and delineation of the plane between the fibroid and myometrium (pseudocapsule) using traction and counter traction should be undertaken to gently enucleate the fibroids. Careful dissection should be taken to minimise the risk of breaching the uterine cavity.
8. Complete repair of the myometrial defect should be effected to avoid leaving ‘dead space’ within the enucleated fibroid bed(s). Care should be taken to avoid passing sutures into the uterine cavity. The edges of the serosal surface should be approximated to prevent exposure of the myometrium. Braided, monofilament or barbed sutures can be used.
9. Patients undergoing laparoscopic myomectomy where power morcellation is to be used to remove the fibroid tissue should be counselled about the small risk of disseminating unexpected malignant material. The use of a containment bag to undertake power morcellation should be considered.
10. Interventions including vasopressin, bupivacaine and epinephrine, misoprostol, peri-cervical tourniquet, tranexamic acid and gelatin-thrombin matrix should be considered to help reduce blood loss during myomectomy.
11. Abdominal myomectomy is associated with post-operative adhesion development that can impact upon subsequent fertility. In addition to atraumatic, precise surgery with minimal blood loss, anti-adhesive agents should be considered to try and reduce the risk of de novo post- operative adhesions although clear evidence of effectiveness is lacking.
12. In cases where fertility is a primary concern and endometrial damage has occurred or been suspected during myomectomy, consider performing a hysteroscopy a minimum of 6 weeks post-operatively to exclude and treat any uterine adhesions.

### What does this mean for patients?

These recommendations have been produced by the European Society for Gynaecological Endoscopy (ESGE) Uterine Fibroids Working Group. The objective of the Working Group was to develop guidance based on the best available evidence and expert opinion for the surgical treatment of uterine fibroids. The recommendations are intended to provide information for clinicians involved in the care of women where surgical treatment of fibroids is indicated because of ongoing pressure, pain or bleeding symptoms or because they interfere with fertility and pregnancy.

The working group has produced two documents describing procedures to perform myomectomy utilising both abdominal (part 1) and hysteroscopic (part 2) routes. The first document (part 1) focuses on abdominal (laparoscopic and open) myomectomy and considers preoperative work-up and intraoperative techniques such as the mode of incision, methods of dissection and suturing and where relevant, morcellation. Postoperative management is also covered with recommendations on convalescence and future pregnancies.
